# Prediction-guided clustering for sepsis phenotyping: a retrospective cohort analysis

**DOI:** 10.1186/s40635-026-00882-9

**Published:** 2026-03-18

**Authors:** Paul A. Hilders, Lada Lijović, Martijn Otten, Laurens A. Biesheuvel, Floor Hiemstra, Marcel van der Kuil, Ameet R. Jagesar, P. J. Thoral, Ari Ercole, Paul W. G. Elbers

**Affiliations:** 1 Department of Intensive Care Medicine, Center for Critical Care Computational Intelligence, Amsterdam Medical Data Science, Amsterdam Public Health, Amsterdam Institute for Immunology and Infectious Diseases, Amsterdam UMC, Vrije Universiteit, University of Amsterdam, Amsterdam, The Netherlands; 2 Quantitative Data Analytics Group, Department of Computer Science, Faculty of Science, Vrije Universiteit, Amsterdam, The Netherlands; 3 Department of Anesthesiology, Intensive Care and Pain Management, University Hospital Center Sestre Milosrdnice, Zagreb, Croatia; 4 Department of Intensive Care Medicine, Leiden University Medical Center, Leiden, The Netherlands; 5 Group of Circadian Medicine, Department of Cell and Chemical Biology, Leiden University Medical Center, Albinusdreef 2, 2333 ZA Leiden, The Netherlands; 6 BBO.Life, Zutphen, The Netherlands; 7 Department of Intensive Care Medicine, OLVG, Amsterdam, The Netherlands; 8 University of Cambridge, Cambridge, UK

**Keywords:** Critical care, Sepsis, Phenotype, Machine learning, Deep learning, Artificial intelligence

## Abstract

**Background:**

Sepsis is a major cause of morbidity and mortality worldwide, with its heterogeneous and dynamically evolving clinical presentation complicating diagnosis, treatment, and prognosis. The identification of clinically meaningful sub-phenotypes within the sepsis population could help tailor interventions and improve outcomes. However, existing phenotyping studies have yielded inconsistent results with limited clinical utility. In this study, we propose a novel, guided machine-learning approach to identify clinically relevant sub-phenotypes within the sepsis condition by integrating deep representation learning with prediction-guided clustering to capture temporal disease trajectories.

**Methods:**

We trained a recurrent neural network-based encoder to generate compact, predictive representations of sepsis patients over time. During training, the encoder is guided by four auxiliary prediction objectives (i.e., 90-day mortality, remaining length of stay, need for mechanical ventilation, and need for renal replacement therapy), which encourage the model to create representations that are relevant with respect to patient-centred outcomes. After training, patient representations were clustered using the *K*-means algorithm. The identified sub-phenotypes were compared across two large ICU data sets (AmsterdamUMCdb and MIMIC-IV) and interpreted using Integrated Gradients-based attribution maps. Practical and clinical utility of the phenotypes was evaluated using a reinforcement learning framework to evaluate optimal treatment strategies within each sepsis sub-phenotype.

**Results:**

Through our approach, we identified six clinically distinct sub-phenotypes with varying risk profiles and presentations. The learned representations demonstrated robust generalisability across the different data sets, and the reinforcement learning results indicated that the different sub-phenotypes were associated with different optimal treatment strategies, highlighting the potential for phenotype-informed decision-making.

**Conclusions:**

This study introduces a flexible and effective framework for the identification of robust and clinically meaningful sub-phenotypes within the population of sepsis patients. Moreover, the identified sub-phenotypes are clinically interpretable, and the proposed trajectory-aware phenotyping approach may support the future development of personalised and precision medicine strategies.

**Supplementary Information:**

The online version contains supplementary material available at 10.1186/s40635-026-00882-9.

## Background

Sepsis remains one of the leading causes of death in the intensive care unit (ICU) and is implicated in one in five deaths globally [1]. This life-threatening condition, defined as organ dysfunction resulting from a dysregulated host response to infection, is responsible for approximately 11 million deaths each year [1, 2]. The immense healthcare and economic burden of sepsis highlights the urgent need for effective and targeted interventions to address this complex and often fatal syndrome [3–5].

Despite improvements in sepsis outcomes over recent years, perhaps attributable to advancements in early diagnosis, fluid resuscitation, timely antibiotic administration, and a better understanding of sepsis pathogenesis, mortality rates remain concerningly high [6, 7]. As no specific sepsis treatment exists, patient management generally relies on early recognition and rapid initiation of antibiotic treatment [7]. Drawing insights from oncology, where deeper understanding of disease sub-types has enabled targeted treatments, recent research suggests that identifying distinct sub-phenotypes within the heterogeneous sepsis population could be a critical step toward developing more precise and effective therapeutic strategies [8].

Although efforts to define sepsis sub-phenotypes have shown promise, the findings have often been inconsistent and inconclusive, reflecting the complexity of the condition and the variability in study methodologies [9, 10]. Nonetheless, the consistent observation of more homogeneous patient sub-groups across studies highlights the potential of this approach to advance sepsis care. The emergence of artificial intelligence (AI) and machine-learning (ML) techniques offers a particularly promising avenue for phenotype discovery, enabling the analysis of large, complex data sets to uncover patterns that may not be discernible through traditional methods [11].

In this study, we propose a novel machine-learning-based approach to identify and evaluate sepsis sub-phenotypes. By leveraging advanced computational methods, we aim to contribute to a deeper, data-driven understanding of sepsis heterogeneity and provide a foundation for future research into personalised treatment strategies. Furthermore, we introduce and demonstrate a modular phenotyping methodology that is highly flexible and designed to generalise easily to other clinical applications, offering a versatile framework for broader translational use.

## Methods

In this section, we describe the methodological framework used to identify and evaluate sepsis sub-phenotypes in the present work. We detail the outline of our guided phenotyping model, including its individual components and the overall clustering network, before providing an explanation on the Integrated Gradients-based attribution maps we use for model interpretability. Finally, we expand upon several methods of evaluation to evaluate the predictive performance, clinical interpretability, and practical utility of the phenotypes that we identify.

### Guided phenotyping approach

Due to the heterogeneous nature of the sepsis population, ‘naive’ and traditional approaches to the clustering of observational clinical data will likely result in clusters based on readily observable attributes, such as commonalities in age, body mass index, and body temperature, rather than sepsis-specific characteristics. This is because unsupervised clustering methods tend to group patients by dominant patterns in the input data, regardless of whether those patterns are clinically relevant to sepsis. This, in turn, leads to the identification of sub-groups that may reflect general demographic or physiological similarities rather than pathophysiological features of the condition. Although these general attributes could, in principle, be based on sepsis-specific patient characteristics or aspects related to relevant clinical outcomes, this would presumably be a product of chance rather than design. As a result, whether the clusters that we identify in the naive approach will represent meaningful sepsis sub-phenotypes, let alone be clinically useful, would generally be outside of our control.

To overcome these limitations, we propose a guided phenotyping framework combining advanced machine-learning techniques with sepsis-related, clinically relevant objectives. At its core, our approach integrates a recurrent neural network-based encoder for enhanced patient representation and a multi-head predictor network to guide meaningful feature extraction.

This framework is designed to summarise a patient’s evolving clinical state over time, rather than to optimise prediction of any single outcome. By incorporating several clinically relevant outcomes during model training, the encoder is encouraged to capture patterns that reflect how different physiological processes develop and interact throughout the course of sepsis. As a result, the subsequent clustering is informed by trajectories of illness over time, instead of relying on isolated measurements or predefined cutoffs. This is particularly relevant for a heterogeneous and dynamically evolving condition, such as sepsis. In this sense, the flexibility and learning capacity of deep learning models serve to complement established clustering approaches by integrating temporal and multi-variable information, rather than to replace simpler phenotyping strategies outright.

In the following subsections, we will further elaborate on the design and purpose of each component within our framework.

#### Encoder component

The encoder network constitutes the backbone of the clustering method, transforming raw, high-dimensional patient data into a compact and contextually enriched representation. This encoded representation is intended to capture complex feature relationships and temporal dynamics in the data, forming the foundation for the downstream prediction and clustering tasks.

Designing the encoder involves critical choices that directly affect the clustering outcomes. For instance, selecting an encoding size smaller than the input dimensionality encourages the model to distil the data, retaining only the most relevant features while discarding noise or less pertinent information. In addition, the choice of a recurrent architecture, such as a long short-term memory (LSTM) [12] model, ensures that temporal dependencies in the variable-length data are preserved, a crucial factor when analysing sepsis progression over time. In practical terms, this allows the model to account for how a patient’s condition evolves over time, rather than analysing isolated measurements. Conversely, non-recurrent architectures may be preferable in cases where temporal patterns are expected to be less relevant.

In this work, we utilise an LSTM-based encoder with an encoding size smaller than the input dimensions to emphasise compact and clinically meaningful representations. A detailed description of the encoder’s architecture, including hyperparameters and training strategies, is provided in Additional file 1: Appendix A.4.

#### Predictor component

The predictor component comprises several prediction networks, each tasked with estimating a distinct clinical outcome relevant to sepsis management. While these predictors are not directly involved in the final clustering phase, they play a crucial role in shaping the encoder to produce meaningful patient representations.

Although large, complex predictor networks would likely allow for enhanced prediction performance, the prediction tasks merely act as a proxy objective while in the process of learning to encode the patient data. By designing the predictors to be relatively simple, we limit their capacity to independently learn complex patterns from the raw data. This constraint encourages the encoder to generate representations that are informative across all prediction tasks, fostering the discovery of shared, clinically relevant features in the encoded data that will eventually be used for clustering.

In this work, we consider the four prediction tasks (a) 90-day mortality, (b) remaining length of stay (LOS), (c) need for mechanical ventilation (MV), and (d) need for renal replacement therapy (RRT).

Predictions for all outcomes are generated at each time step based on the patient’s encoded representation at that point in time, rather than on cluster membership itself. As a result, predicted risks may evolve over time as new clinical information becomes available, and patients assigned to the same sub-phenotype at different stages of their ICU course may have different predicted outcomes. Importantly, the prediction tasks are not intended as stand-alone clinical forecasting models or to distinguish explicitly between treatment initiation and continuation. Instead, they serve as guiding signals to ensure that the learned representations encode clinically relevant information related to disease severity, organ support, and evolving disease course.

#### Combined clustering network

The proposed clustering framework unfolds in two stages. In the initial stage, we focus on training the encoder component to generate enhanced patient representations. In the second phase, we use the trained encoder to cluster our patients based on the generated encoded representations.

**Fig. 1:**
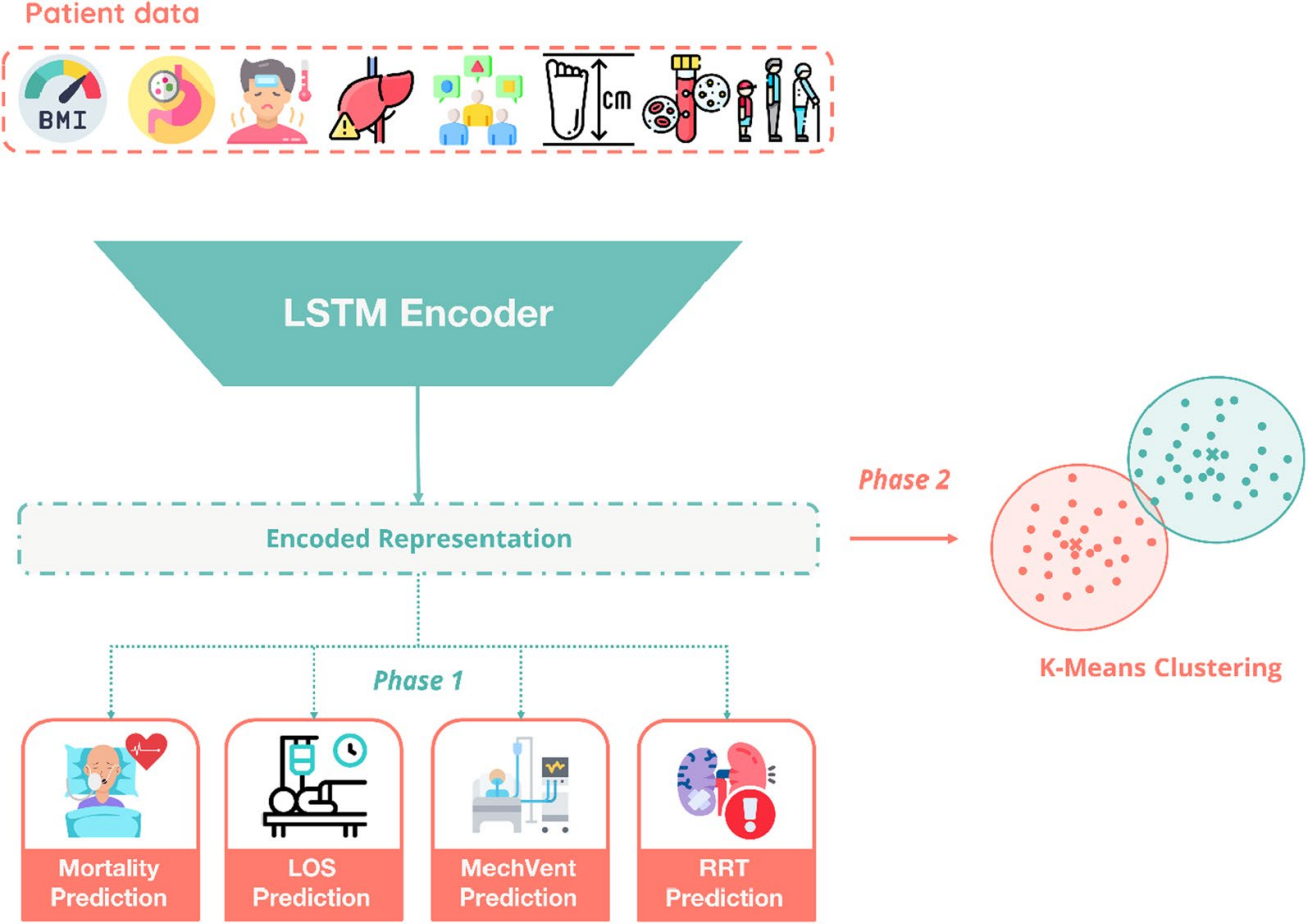
Visualisation of the combined clustering network. LOS: length of ICU stay, MechVent: mechanical ventilation, RRT: renal replacement therapy, LSTM: long short-term memory

During training, the encoder and predictor components of the combined network (Fig. 1) are trained in an end-to-end fashion. At every update step, the loss for each prediction task is computed, weighted, and aggregated to form a single loss value. The combined loss is then backpropagated through the entire network, and the training process continues until the aggregated validation loss reaches convergence.

In the second phase, the trained encoder is employed to transform the patient data from the validation set into encoded representations. These representations serve as input to a clustering algorithm, such as *K*-means, which groups patients into distinct sub-phenotypes. To determine the optimal number of clusters, we use silhouette analysis, which evaluates the cohesion and separation of clusters. This two-stage approach ensures that the clustering results are both data-driven and clinically interpretable.

### Model explainability: Integrated Gradients-based attribution maps

Once the patient encodings have been categorised with the clustering algorithm, the next objective is to compare and label the resulting clusters. Unfortunately, cluster comparison solely based on sample statistics can only provide limited information. It would be very challenging, if not impossible, to determine whether cluster differences are coincidental or if they actually had a meaningful effect on the generated encoding.

Therefore, model interpretability is a crucial component of our framework, to enhance the trustworthiness and reliability of the identified sub-phenotypes. In the present work, we employ Integrated Gradients-based attribution maps, a technique that quantifies the contribution of individual input features to the output encodings [13]. Whereas the cluster sample statistics inform us how two sub-phenotypes differ, the attribution maps can inform us which differences had a meaningful impact on the encodings that were used for clustering.

### Methods of evaluation

Evaluation of a phenotyping model is inherently challenging due to the complexity and multi-faceted nature of the sepsis condition. Intuitively, an ideal evaluation would demonstrate that the identified sub-phenotypes reflect meaningful clinical patterns, aligning with existing knowledge of sepsis pathophysiology while also offering novel insights. However, this alignment must be approached cautiously to avoid confirmation bias, where results are interpreted solely through the lens of prior expectations.

To mitigate these risks, we employ a multi-faceted evaluation strategy. This includes assessing the predictive performance of the model, examining the clinical interpretability of the sub-phenotypes, and exploring their potential utility in practical settings. In addition, we will examine the generalisability of the sub-phenotypes by performing external evaluation. Through this approach, we aim to provide a balanced and objective appraisal of their validity and relevance. Even so, we strongly encourage readers to deliberate the results critically and independently, to ensure that the sub-phenotypes are not merely evaluated based on preconceived notions.

#### Practical utility: a reinforcement learning-based assessment

To evaluate the practical utility of the identified sub-phenotypes, we will explore their potential impact on clinical decision-making using a reinforcement learning-based approach, inspired by [14]. Specifically, we employ the state–action–reward–state–action (SARSA) algorithm [15] to estimate the Q-function for the behaviour policy that generated the data. In non-technical terms, this approach allows us to quantify the effectiveness of the treatment strategies employed by the physicians in our data set and assess how these strategies have influenced clinical outcomes. In contrast to other traditionally on-policy reinforcement learning algorithms, estimating the physician value function using a SARSA-style approach allows us to simply evaluate the merits of the decision-making process reflected in our data sets, across our defined action and state space (i.e., the range of identified sub-phenotypes), without considering out-of-distribution actions.

This experiment serves two primary purposes. First, it evaluates the extent to which sub-phenotypes can inform tailored interventions, potentially leading to improved patient outcomes. Second, it demonstrates how the identification and characterisation of novel sub-phenotypes can catalyse future research into optimising sepsis treatment strategies. The resulting Q-function offers a nuanced understanding of the clinical decision-making process. It not only reflects how specific treatment combinations have historically impacted patient trajectories within each sub-phenotype but also highlights areas, where certain treatment strategies may have been underexplored. Consequently, the application of reinforcement learning within the space of sub-phenotypes identified in this study allows us to assess the potential benefits of phenotype-informed decision-making in a controlled, reproducible setting, offering valuable insights into their real-world applicability.

## Data

The primary data set in this study was extracted from AmsterdamUMCdb, a publicly accessible database consisting of de-identified health data from 23,106 admissions to the intensive care unit (ICU) of Amsterdam University Medical Centre in the Netherlands between 2003 and 2016 [16]. The secondary validation data set was extracted from the Medical Information Mart for Intensive Care IV (MIMIC-IV) database, which contains de-identified medical data for over 65,000 patients admitted to an ICU at the Beth Israel Deaconess Medical Centre between 2008 and 2019 [17]. This secondary data set enabled us to assess the generalisability of our prediction performance and the consistency of identified clusters across diverse patient populations.

All adult, critically ill patients who fulfilled the criteria of the Sepsis-3 definition [2] within 24 h after admission to the ICU were deemed eligible for inclusion. For these patients, we extracted clinically routinely collected variables which could potentially be informative of relevant clinical outcomes related to sepsis. Data were summarised into distinct time steps by aggregating values over 4-h intervals. Based on domain knowledge and the availability of data across both data sets, the following features were selected:

- Demographics (4): Admission age, weight, height, and gender.
- Laboratory values (22): Albumin, aspartate transaminase (AST), alanine transaminase (ALT), bicarbonate, bilirubin, blood urea nitrogen (BUN), calcium, chloride, potassium, C-reactive protein (CRP), creatinine, glucose, hematocrit, hemoglobin, international normalised ratio (INR), lactate, partial pressure of oxygen (PaO_2_), partial pressure of carbon dioxide (PaCO_2_), pH, platelets, sodium, and white blood cell count (WBC).
- Vital signs (8): Heart rate, oxygen saturation (SpO_2_), respiratory rate, systolic blood pressure, mean blood pressure, diastolic blood pressure, central venous pressure (CVP), and temperature.
- Other clinical measures (5): Glasgow coma scale (GCS), positive end-expiratory pressure (PEEP), documented renal replacement therapy (RRT), administration of intravenous (IV) fluids, and vasopressor use (converted to norepinephrine equivalents).

To create summary metrics for analysis, we calculated the minimum GCS score, maximum PEEP value, maximum vasopressor rate, total IV fluid volume, and the average values for all remaining features within each time window. After data extraction, we first performed outlier detection and removal based on handpicked value thresholds, determined by clinical expertise. Then, we used forward-filling to propagate known measurements forward in time for a maximum of 24 h, to account for measurement gaps. After, we excluded features with more than 60% missing measurements and patients with more than 30% missing data. Finally, we used median imputation to fill in the missing values for the remainder of the incomplete features, and added two binary labels to represent missing values for width and height before imputation.

After data processing, we defined the final predictor objectives based on clinically relevant outcomes: (a) 90-day mortality, (b) remaining length of stay, (c) documented next-time step renal replacement therapy, and (d) next-time step mechanical ventilation, inferred from documented PEEP settings.

In addition, vasopressor infusion rates and the cumulative volume of IV fluids administered at each time step were used to define the action space for the reinforcement learning-based experiment in a similar fashion to [14]. These variables were excluded from the input space of the clustering model to avoid confounding the sub-phenotype representation with direct treatment interventions.

## Results

### Data sets

After processing, the data sets were divided into a training, validation, and test data set using a random 70–15–15% split. The resulting data set statistics are shown in Table 1. Based on our previously specified exclusion criteria, we excluded 340 patients (18.1%) from the AmsterdamUMCdb cohort and 4944 patients (15.0%) from the MIMIC-IV cohort due to an insufficient number of measurements. In addition, we excluded albumin, CRP, and CVP from our feature set due to limited availability in either of the two data sets, leading to the final feature selection displayed in Table 1.

**Table 1:** Overview of data set statistics after preprocessing

Data set	No. of admissions				No. of time steps			
	Train	Val	Test	Total	Train	Val	Test	Total
AmsterdamUMCdb	1073	231	231	1535	102,768	23,616	19,629	146,013
MIMIC-IV	19,633	4208	4208	28,049	692,132	146,026	146,103	984,261

### Prediction performance

Table 2 presents the prediction performance of the model trained on the AmsterdamUMCdb training set across all prediction tasks for both the AmsterdamUMCdb test set and the full MIMIC-IV data set. When evaluated on the AmsterdamUMCdb test set, the model achieved an area under the receiver operating characteristic (AUROC) score of 0.736 for predicting 90-day mortality, 0.883 for next-time step renal replacement therapy (RRT), and 0.723 for next-time step mechanical ventilation (MV). For the length-of-stay (LOS) prediction task, the model recorded a mean squared error (MSE) of 118.04.

**Table 2:** Model prediction performance across the four sepsis-related prediction tasks for the test set from AmsterdamUMCdb and the full MIMIC-IV data set

Performance	AmsterdamUMCdb (Test set)		MIMIC-IV (Full data set)	
90-day mortality (Accuracy|AUROC)	0.667 (0.612–0.743)	0.736 (0.652–0.818)	0.711 (0.705–0.719)	0.713 (0.704–0.724)
Length of stay (mean squared error)	118.04 (63.53–205.57)		32.61 (32.00–33.31)	
RRT (Accuracy|AUROC)	0.861 (0.803–0.913)	0.883 (0.841–0.920)	0.929 (0.926–0.931)	0.906 (0.900–0.912)
MV (Accuracy|AUROC)	0.710 (0.668–0.751)	0.723 (0.677–0.769)	0.576 (0.571–0.580)	0.614 (0.608–0.618)

Values are mean (95% CI). *AUROC* area under the receiver operating characteristic curve

When applied to the external validation data set from the full MIMIC-IV cohort, the model demonstrated AUROC scores of 0.713, 0.906, and 0.614 for the 90-day mortality, RRT, and MV prediction tasks, respectively, while achieving an MSE of 32.61 for LOS prediction.

### Clustering results

After training the model on the AmsterdamUMCdb training set, clustering was performed on the validation data set using the K-means algorithm. Due to computational constraints, clustering on the MIMIC-IV data set was limited to a 15% subset of the data, mirroring the proportion used for clustering in the AmsterdamUMCdb validation set. Using silhouette analysis, we identified the optimal number of *K*-means clusters as six (Additional file 1: Appendix A.6). Table 3 presents the distribution of data points across the clusters, along with the average feature values and their standard deviations. These statistics reveal numerous inter-cluster differences that may be clinically meaningful. To further investigate these differences and their implications, we employed the Integrated Gradients-based explainability technique.

**Table 3:** Sample statistics across the six clusters from the AmsterdamUMCdb validation set

Features	Cohort	A0	A1	A2	A3	A4	A5
*Number of time steps, N (%)*	23,616	3659 (15.5%)	682 (2.9%)	4491 (19.0%)	1355 (5.7%)	5618 (23.8%)	7811 (33.1%)
*AST, U/L*	173.7 (± 113.3)	246.4 (± 1163.3)	178.8 (± 443.1)	157.1 (± 630.7)	348.8 (± 1446.0)	51.1 (± 76.9)	60.2 (± 145.1)
*ALT, U/L*	132.2 (± 53.6)	190.5 (± 701.7)	136.6 (± 242.7)	158.4 (± 516.3)	173.8 (± 414.5)	62.7 (± 98.1)	71.3 (± 145.7)
*Bicarbonate, mmol/L*	25.6 (± 1.7)	24.1 (± 5.1)	25.3 (± 4.7)	25.5 (± 4.7)	23.7 (± 5.9)	27.7 (± 3.9)	27.4 (± 4.3)
*Total bilirubin, µmol/L*	19.7 (± 13.0)	13.2 (± 17.2)	20.8 (± 30.0)	15.0 (± 20.0)	44.9 (± 79.6)	8.3 (± 3.3)	16.0 (± 27.1)
*BUN, mmol/L*	11.7 [11.7,11.8]	11.3 [6.9,12.9]	11.9 [11.7,20.2]	11.7 [11.1,13.1]	12.9 [11.3,21.7]	11.7 [8.4,11.7]	11.7 [11.0,14.8]
*Calcium, mmol/L*	2.03 (± 0.04)	1.98 (± 0.19)	2.02 (± 0.16)	2.01 (± 0.20)	2.02 (± 0.24)	2.08 (± 0.17)	2.09 (± 0.20)
*Chloride, mmol/L*	104.9 (± 1.0)	106.0 (± 5.4)	104.2 (± 2.7)	105.7 (± 4.8)	105.6 (± 6.6)	103.6 (± 3.8)	104.1 (± 4.6)
*Potassium, mmol/l*	4.2 (± 0.1)	4.1 (± 0.5)	4.2 (± 0.5)	4.2 (± 0.4)	4.3 (± 0.6)	4.1 (± 0.3)	4.2 (± 0.4)
*Serum creatinine, µmol/L*	93.0 [84.8, 108.0]	83.0 [57.0, 133.0]	234.0 [190.0, 318.0]	96.0 [73.0, 165.0]	112.0 [78.0, 156.5]	57.0 [42.0, 80.0]	90.0 [66.0, 149.0]
*Blood glucose, mmol/L*	6.9 [6.8,7.2]	7.7 [6.6,9.0]	6.9 [5.9,8.4]	7.3 [6.3,8.5]	6.8 [5.8,8.0]	6.9 [6.2,7.8]	6.8 [6.0,7.7]
*Heart rate, bpm*	90.6 (± 2.5)	89.9 (± 19.5)	90.9 (± 18.8)	89.1 (± 17.3)	95.5 (± 19.7)	90.0 (± 16.7)	88.4 (± 16.7)
*Hematocrit, fraction*	0.288 (± 0.016)	0.315 (± 0.049)	0.276 (± 0.037)	0.297 (± 0.041)	0.280 (± 0.034)	0.285 (± 0.036)	0.274 (± 0.030)
*Hemoglobin, g/L*	5.9 (± 0.3)	6.4 (± 1.0)	5.7 (± 0.8)	6.1 (± 0.9)	5.7 (± 0.7)	5.8 (± 0.8)	5.6 (± 0.7)
*INR*	1.3 [1.2,1.3]	1.3 [1.2,1.5]	1.3 [1.2,1.4]	1.2 [1.2,1.4]	1.6 [1.2,2.1]	1.2 [1.2,1.2]	1.2 [1.2,1.4]
*Lactate, mmol/L*	1.8 (± 0.6)	1.8 (± 1.6)	1.9 (± 1.8)	1.6 (± 1.3)	2.9 (± 3.4)	1.3 (± 0.3)	1.5 (± 0.6)
*SpO*_2_, %	97.8 [96.9,98.0]	96.5 [95.0,98.2]	98.0 [96.7,99.0]	97.7 [96.0,99.0]	96.7 [95.0,98.5]	98.0 [96.2,99.2]	98.0 [96.7,99.3]
*PaO*_2_, mmHg	91.5 [88.0, 92.8]	86.0 [72.5, 105.0]	91.0 [79.0, 105.0]	92.0 [78.5, 110.0]	87.0 [74.0, 104.2]	93.0 [81.0, 114.0]	93.0 [80.0, 113.0]
PaCO_2_, mmHg	41.5 [40.2,43.5]	39.0 [33.5,44.0]	42.0 [37.3,45.0]	40.0 [35.0,45.0]	44.0 [39.0,51.0]	41.0 [36.0,46.0]	44.0 [39.0,50.0]
*Arterial pH*	7.41 [7.40,7.43]	7.41 [7.35,7.45]	7.40 [7.36,7.44]	7.43 [7.37,7.46]	7.34 [7.27,7.39]	7.45 [7.42,7.48]	7.42 [7.37,7.45]
*Platelets*	213.2 [196.2, 235.1]	238.0 [153.0, 352.0]	226.5 [123.0, 409.0]	195.0 [113.0, 303.0]	70.0 [42.0, 100.0]	391.0 [285.0, 513.0]	200.0 [124.0, 311.5]
*Respiratory rate, per minute*	21.8 (± 0.9)	20.8 (± 6.2)	20.9 (± 4.2)	21.7 (± 4.6)	22.5 (± 5.6)	22.4 (± 5.1)	22.8 (± 4.8)
*Sodium, mmol/L*	140.1 (± 0.8)	139.5 (± 5.9)	141.3 (± 6.4)	140.9 (± 5.6)	139.5 (± 6.4)	139.5 (± 4.9)	140.0 (± 6.4)
*Systolic BP, mmHg*	129.6 (± 9.5)	125.1 (± 22.6)	138.4 (± 26.0)	136.5 (± 24.4)	112.7 (± 19.5)	134.1 (± 22.0)	130.8 (± 21.7)
*Mean BP, mmHg*	82.4 (± 5.9)	83.0 (± 13.8)	87.1 (± 13.3)	85.5 (± 13.7)	71.7 (± 9.6)	87.0 (± 13.5)	80.3 (± 13.0)
*Diastolic BP, mmHg*	60.1 (± 4.7)	61.9 (± 11.3)	63.9 (± 9.9)	62.1 (± 11.0)	52.5 (± 8.7)	63.9 (± 11.5)	56.2 (± 11.0)
*Temperature (°C)*	36.8 (± 0.2)	36.7 (± 1.0)	37.0 (± 0.9)	37.0 (± 0.9)	36.6 (± 0.9)	37.0 (± 0.8)	36.7 (± 0.8)
*WBC. × 10*^9^/*L*	13.1 [12.5,13.2]	13.3 [9.5,17.8]	13.1 [10.7,16.9]	13.1 [9.3,18.5]	11.9 [6.2,18.8]	12.3 [9.4,15.3]	13.7 [10.2,19.5]
*GCS eye*	3.5 (± 0.2)	3.7 (± 0.8)	3.6 (± 0.9)	3.1 (± 1.2)	3.3 (± 1.1)	3.6 (± 1.0)	3.7 (± 0.7)
*GCS motor*	5.2 (± 0.4)	5.7 (± 1.1)	5.1 (± 1.9)	4.7 (± 1.9)	4.9 (± 2.0)	5.4 (± 1.3)	5.3 (± 1.6)
*GCS verbal*	1.8 (± 0.7)	3.0 (± 1.9)	1.8 (± 1.6)	1.4 (± 1.2)	1.5 (± 1.2)	2.1 (± 1.7)	1.2 (± 0.8)
*Age (norm)*	0.29 (± 0.15)	0.27 (± 0.41)	0.10 (± 0.24)	0.29 (± 0.50)	0.41 (± 0.33)	0.19 (± 0.54)	0.51 (± 0.34)
*Weight (norm)*	− 0.16 (± 0.34)	− 0.16 (± 0.48)	0.43 (± 0.63)	− 0.11 (± 0.50)	− 0.59 (± 0.39)	− 0.13 (± 0.52)	− 0.36 (± 0.55)
*Height (norm)*	− 0.05 (± 0.13)	0.01 (± 0.47)	− 0.02 (± 0.18)	0.08 (± 0.46)	− 0.18 (± 0.35)	0.06 (± 0.50)	− 0.24 (± 0.40)
*Weight missing, N* (%)	1092 (4.6%)	43 (1.2%)	0 (0.0%)	140 (3.1%)	265 (19.6%)	17 (0.3%)	282 (3.6%)
*Height missing, N* (%)	1171 (5.0%)	49 (1.3%)	0 (0.0%)	206 (4.6%)	265 (19.6%)	17 (0.3%)	311 (4.0%)
*Gender (male), N* (%)	13,870 (58.7%)	2159 (59.0%)	543 (79.6%)	2892 (64.4%)	571 (42.1%)	3758 (66.9%)	3152 (40.4%)
*90-day mortality, N* (%)	9240 (39.1%)	1009 (27.6%)	60 (8.8%)	1448 (32.2%)	1090 (80.4%)	1596 (28.4%)	4476 (57.3%)
*LOS outcome, days*	13.0 [10.1,18.7]	6.5 [2.4,16.6]	23.9 [5.3,53.1]	14.2 [6.6,31.7]	11.7 [3.5,33.3]	9.5 [4.0,19.0]	20.1 [8.4,37.1]
*RRT outcome, N* (%)	4470 (18.9%)	179 (4.9%)	198 (29.0%)	604 (13.4%)	483 (35.6%)	123 (2.2%)	2216 (28.4%)
*MV outcome, N* (%)	16,601 (70.3%)	1903 (52.0%)	502 (73.6%)	3680 (81.9%)	1203 (88.8%)	2680 (47.7%)	6073 (77.7%)

Values are mean(± SD), median[Q1,Q3] or *N* (%). *AST* aspartate aminotransferase, *ALT* alanine aminotransferase, *BUN* blood urea nitrogen, *INR* international normalized ratio, *BP* blood pressure, *WBC* white blood cell count, *GCS* Glasgow coma score, *LOS* length of stay, *RRT* renal replacement therapy, *MV* mechanical ventilation

#### Integrated Gradients-based attribution maps

**Fig. 2:**
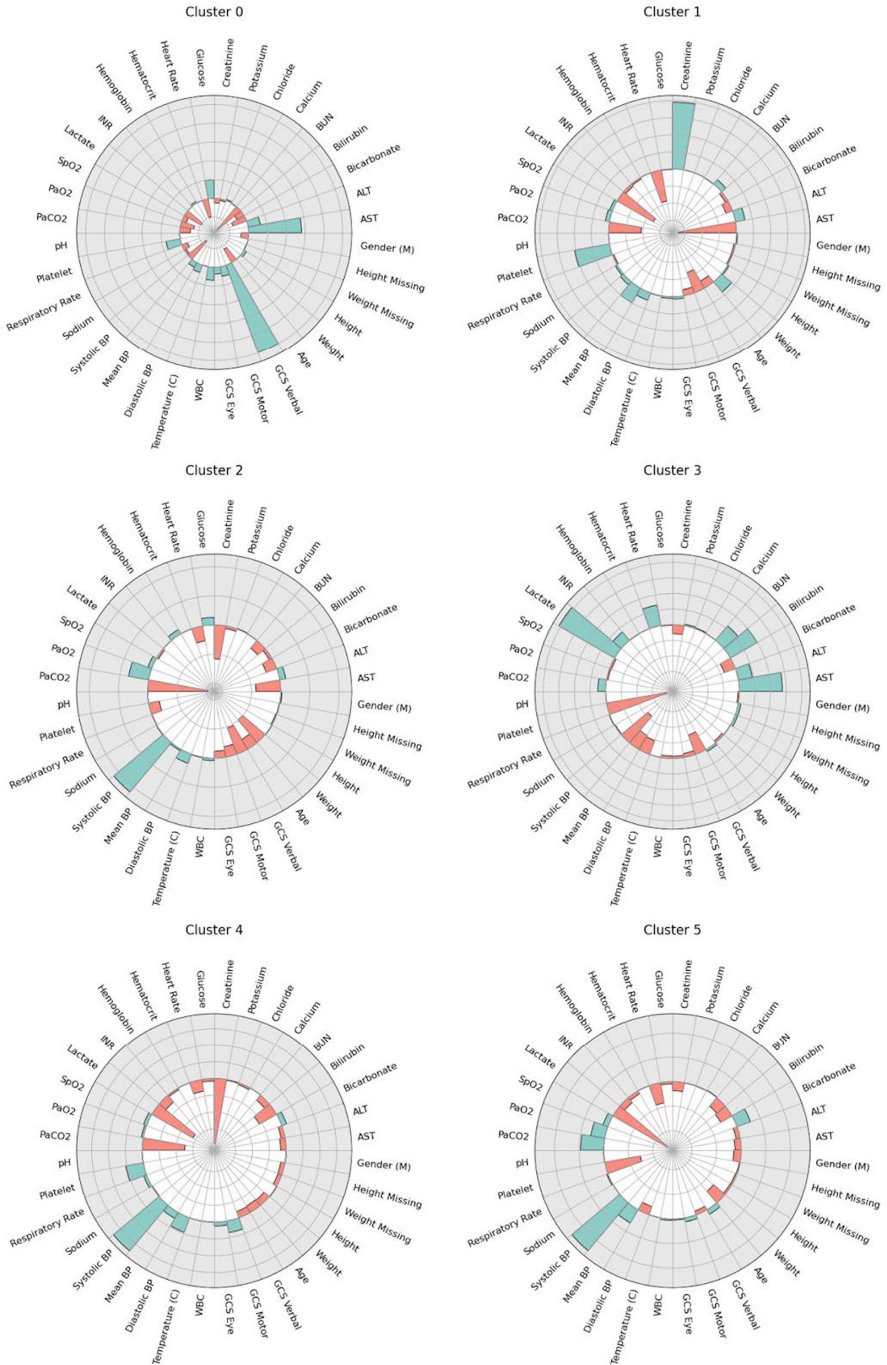
Visualisation of Integrated Gradients-based attribution values across the various features and clusters in the AmsterdamUMCdb data set. A larger value or bar size represents a relatively large contribution of that feature to the average encoding that was assigned to the corresponding cluster. The colour and directionality of the bar denote a high (green/outside) or low (red/inside) average feature value, relative to the other clusters

The attribution maps in Fig. 2 illustrate how individual features, on average, influenced the encoded representations assigned to each cluster. Specifically, a large attribution value (indicated by the bar size) reflects a relatively strong impact of a feature on the average encoding for the respective cluster. The bar colours represent the relative values of the features, where green denotes higher average feature values and red denotes lower average feature values, compared to the data set mean.

#### Sub-phenotype specification

In this subsection, we integrate insights from the sample statistics (Table 3 and Additional file 1: Table A2), attribution maps (Fig. 2 and Additional file 1: Fig. A3), and Sankey diagrams (Additional file 1: Appendix A1) to describe the characteristics and defining properties of the six identified sub-phenotypes for both AmsterdamUMCdb and MIMIC-IV.

**Fig. 3:**
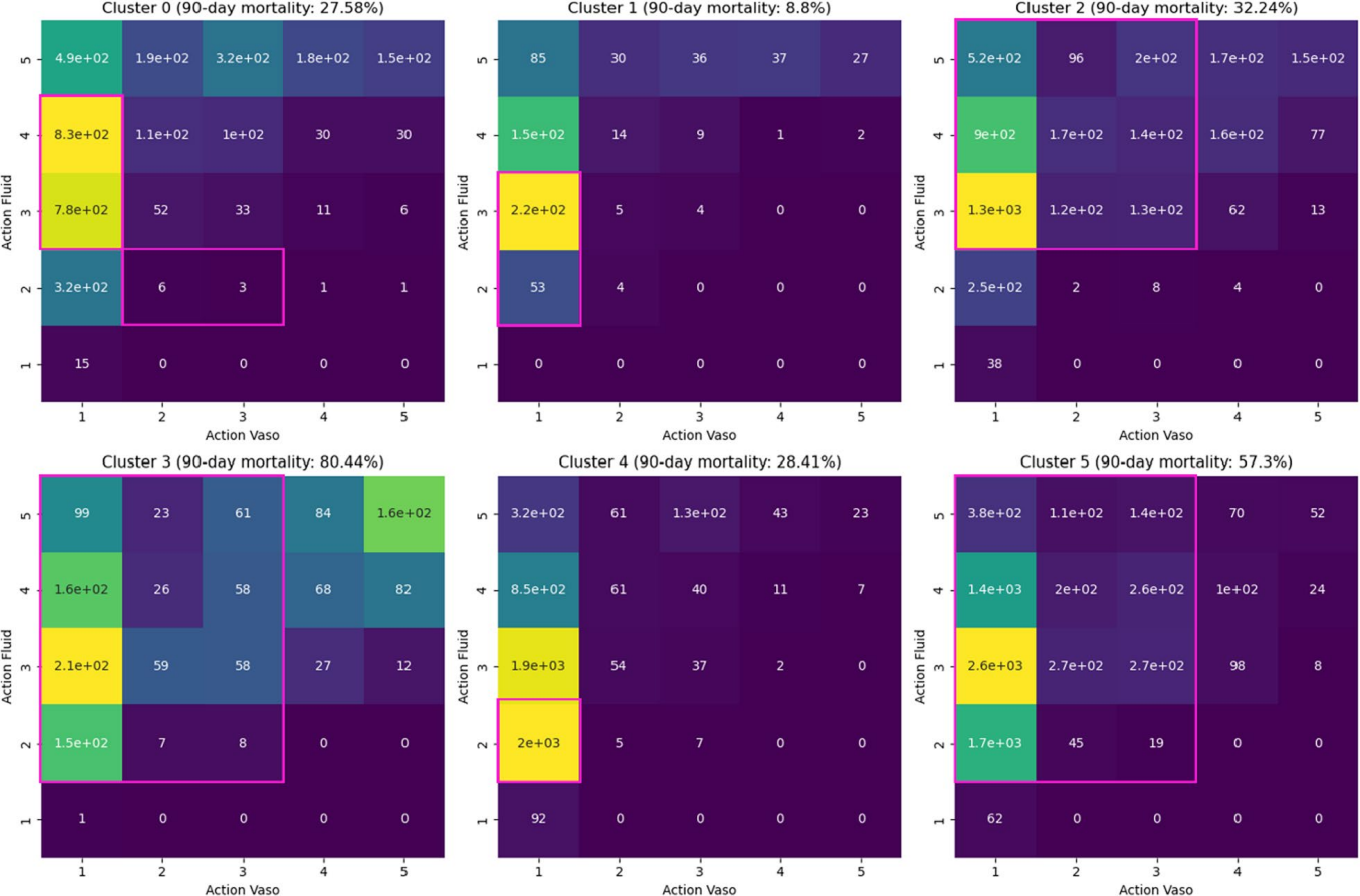
Visualisation of the observed distribution of treatment strategies across the six sub-phenotypes in the AmsterdamUMCdb cohort. The horizontal axis (“Action Fluid”) represents discretised levels of administered intravenous fluid volume, while the vertical axis (“Action Vaso”) represents discretised vasopressor intensity expressed as norepinephrine equivalents. Colour indicates how frequently a treatment combination was observed in the data set (yellow = frequent; dark purple = rare). The purple bounding boxes highlight decision regions estimated to provide the most favourable outcomes according to the SARSA reinforcement learning algorithm

##### AmsterdamUMCdb sub-phenotypes

- Cluster A0 represents an early stage of sepsis in the ICU, with moderate physiological derangements but relatively preserved haemodynamics and higher GCS verbal scores compared with other clusters. It is most prevalent early in the trajectory and serves as a starting point from which patients diverge into more favourable (A4) or more severe (A2, A3, A5) states.
- Cluster A1 represents a relatively small (2.9%) renal-dominant phenotype. It is characterised by the highest serum creatinine levels and a high prevalence of renal replacement therapy, while blood pressure, lactate, and other organ-specific markers are comparatively preserved. Despite severe acute kidney injury and longer ICU stays, phenotype A1 has the lowest 90-day mortality rate (8.8%), suggesting a subset with substantial but potentially reversible renal dysfunction.
- Cluster A2 primarily includes mechanically ventilated patients and patients with relatively high systolic blood pressures, moderate lactate and creatinine levels, and an intermediate mortality rate. Phenotype A2 reflects an early respiratory-failure state in which pulmonary support is prominent, whereas circulatory shock and renal or hepatic failure are less prevalent.
- Cluster A3 contains the most critically ill patients and has the highest 90-day mortality rate (80.4%). Key characteristics include low blood pressure, high AST/ALT (De Ritis) ratio, thrombocytopenia, high lactate, and high bilirubin, indicating severe organ dysfunction and metabolic derangement. Mechanical ventilation and renal replacement therapy are common, consistent with fully developed shock states.
- Cluster A4 represents patients whose clinical parameters have partially normalised during later stages of the ICU stay. This cluster is characterised by low creatinine, lactate, and PaCO_2_, along with high systolic blood pressure and platelets. Its prevalence increases over time, suggesting recovery trajectories.
- Cluster A5 consists of patients who have partially recovered at later stages, often transitioning from Cluster A3. Considering the 57.3% mortality rate, which is significantly higher than that of Cluster A4 (28.4%), this cluster presumably reflects partial improvement with persistent risk rather than a true recovery trajectory.

##### MIMIC-IV sub-phenotypes

- Cluster M0 appears to contain patients with relatively unremarkable values, making eventual outcomes difficult to predict. Cluster M0 is most prevalent in the middle of the ICU stay and the primary influx clusters are M5 and M3, which are both most prevalent at the start of the trajectory. Therefore, Cluster M0 may serve as an intermediate state, from where patients can transition to more or less favourable clusters.
- Cluster M1 contains patients who appear to have partially stabilised at later stages in the ICU stay, becoming more prevalent over time. It is characterised by relatively high blood pressure, low lactate, low bilirubin, low BUN, low RRT, and low mortality.
- Cluster M2 seems to consist of patients who suffer from kidney failure or Acute Kidney Injury (AKI), as this cluster is characterised by relatively high creatinine and the highest prevalence of renal replacement therapy.
- Cluster M3 represents patients who improve early in the ICU stay. The values appear to have mostly recovered and it has the lowest 90-day mortality rate. Cluster M3 is characterised by low BUN, low creatinine, low lactate, low AST, low ALT, and relatively high blood pressure.
- Cluster M4 consists of the most critically ill patients, at any stage of the ICU stay. It is characterised by high BUN, high bilirubin, high AST/ALT (De Ritis) ratio, and low blood pressure. Moreover, it has the highest MV rate, a high RRT usage, and the highest mortality rate (42.7%).
- Cluster M5 reflects the starting point of the ICU trajectory, with patients transitioning into more or less favourable clusters as their condition evolves.

##### Sub-phenotype alignment

Given that the sub-phenotypes were generated independently for each data set, it is noteworthy to explore their similarities and differences. Table 4 shows the clusters for both data sets, along with a description of the corresponding sub-phenotype. Where possible, we aligned clusters with largely overlapping characteristics across data sets and labelled the overarching sub-phenotype accordingly.

**Table 4:** Overview of the identified sub-phenotypes for the AmsterdamUMCdb and MIMIC-IV cohorts, along with a description

AmsterdamUMCdb	MIMIC-IV	Sub-phenotype description
*Cluster A0*	*Cluster M5*	Start of the ICU stay
*Cluster A1*	*Clusters M2, M3*	AKI and/or early improvement in the ICU stay
*Cluster A2*		Patients on mechanical ventilation at an early stage
*Cluster A3*	*Cluster M4*	Most critically ill patients, high risk of mortality
*Cluster A4*	*Cluster M1*	Patients who partially stabilise at a later stage
*Cluster A5*		Patients who partially stabilise after being in the ‘most critically ill’ cluster
	*Cluster M0*	‘Unremarkable’ patients, whose outcome is hard to anticipate

### Practical utility: reinforcement learning results

In this section, we present the results of the reinforcement learning experiment designed to explore the practical utility of the identified sub-phenotypes in guiding clinical decision-making. Using the SARSA algorithm, we explored the treatment strategies employed by physicians in the AmsterdamUMCdb data set across the state space of clusters and action space as defined in Additional file 1: Table A5 [14]. The visualisations in Fig. 3 illustrate the decision regions, with purple bounding boxes highlighting areas identified as optimal based on the learned *Q* values.

The results reveal notable differences in the size and variability of decision regions among clusters. For example, the decision regions for Clusters A3 and A5 are considerably larger, suggesting a broader range of effective treatment strategies compared to Clusters A1 and A4, where the regions are more constrained. Furthermore, while the data indicate that physicians predominantly favour low vasopressor levels combined with varying fluid volumes, the SARSA-derived Q-function suggests a more diverse treatment approach tailored to the characteristics of specific sub-phenotypes.

Crucially, the *Q* values differ not only in magnitude but also in their spatial distribution across the action space for each cluster. This suggests that different combinations of treatment, such as specific pairings of fluid volume and vasopressor dosage, lead to the highest estimated reward in different clusters. In other words, the analysis suggests that estimated treatment–outcome associations differ across sub-phenotypes, indicating that treatment effects may not be homogeneous across the sepsis population.

Taken together, these findings highlight the heterogeneity of the sepsis population and illustrate how reinforcement learning can serve as an exploratory tool to reveal subgroup-specific patterns in treatment decisions and outcomes that merit further evaluation.

## Discussion

In this study, we proposed a guided machine learning-based phenotyping approach to identify and evaluate sub-phenotypes within the population of sepsis patients. Our method integrates a recurrent neural network-based encoder with multiple clinically relevant prediction tasks to generate informative patient representations, which were subsequently clustered using *K*-means. We identified six distinct sub-phenotypes in both the AmsterdamUMCdb and MIMIC-IV cohorts, with notable differences in clinical characteristics, outcomes, and predicted response to treatment strategies.

While the primary aim of this study is to explore clustering outcomes, the model’s solid predictive performance on the test set and its generalisability across an independent data set suggest that the generated encodings capture clinically relevant and meaningful information. This supports the hypothesis that the learned representations are well-suited for characterising the sepsis population, which is essential for downstream clustering and analysis. The approach aligns with recent developments in the field, where machine learning and deep representation learning have been proposed as promising tools for the discovery of clinically relevant sub-phenotypes [18].

Efforts to stratify sepsis into sub-phenotypes have a well-established basis in the literature. Prior studies have identified subgroups based on body temperature trajectories [19], infection sites [20], and hemodynamic profiles, such as systolic blood pressure trends [21]. Latent profile analysis (LPA) and latent class analysis (LCA) on large ICU databases like MIMIC-III have yielded robust phenotypes associated with mortality and organ dysfunction [22, 23]. However, the variability in the axes of comparison, the number of subgroups, and the definitions used within these studies often hampers reproducibility and clinical translation [24]. Against this backdrop, our method seeks to advance the field by generating sub-phenotypes through a guided machine-learning approach, by integrating deep representation learning with prediction-guided clustering to promote clinical relevance and robustness. In contrast to LPA, LCA, and trajectory-based clustering methods, which typically identify latent subgroups based solely on co-occurrence patterns or temporal dynamics, our guided approach explicitly incorporates clinical outcomes into the representation learning process. This allows the resulting phenotypes to be not only statistically distinct but also optimised for relevance to prognosis and potential therapeutic response.

Using Integrated Gradients-based attribution maps allowed us to interpret the sample statistics from Table 3 through the perspective of our learned encoder model, instead of our own potentially biased expectations. For example, while Cluster A3 exhibits low platelet levels and a high proportion of missing height and weight relative to other clusters, the attribution map for this cluster indicates that missing height and weight did not significantly affect the patient representations. Conversely, thrombocytopenia emerged as a defining characteristic of this sub-phenotype, highlighting its potential clinical relevance.

To place these findings in the context of prior sepsis phenotyping studies, we observed several coherent convergences despite methodological and temporal differences. In particular, the A3 cluster (and the analogous M4 cluster) could be considered a trajectory-based analogue of the SENECA δ phenotype, characterised by the lowest mean blood pressure, lowest bicarbonate, highest lactate, similar coagulation patterns, and the highest mortality. In this study, this phenotype is additionally marked by higher levels of organ support, consistent with the approach of capturing ICU trajectories rather than admission data. Notably, although mortality was not included as a clustering variable and only served as a guiding proxy objective during representation learning, this trajectory-based shock phenotype nonetheless emerges with the highest observed mortality, mirroring the SENECA *δ* class. Beyond this *δ*-like shock phenotype, our trajectory-based clusters also show alignment with the remaining SENECA classes. Cluster A4 corresponds most closely to the SENECA α phenotype, capturing relatively preserved or recovering patients with near-normal organ-function markers, low lactate, preserved platelet counts and haemodynamics, and minimal organ support. Clusters A1 and A5 together occupy the *β* spectrum: A1 reflects an early, haemodynamically stable acute kidney-failure state with very high creatinine levels and frequent renal replacement therapy but limited extra-renal dysfunction, whereas A5 represents a later, high-risk chronic critical illness state characterised by persistent renal dysfunction, older age, and high 90-day mortality. Cluster A2 partially parallels the γ phenotype as an early respiratory-failure state with frequent mechanical ventilation and relatively preserved circulation, although inflammatory markers are less specific and lung failure often co-occurs with other organ dysfunctions. A key distinction from the SENECA framework is that our renal phenotypes (A1 vs. A5) separate early potentially reversible acute kidney injury from late chronic critical illness, a level of granularity that cannot be captured from a single early timepoint and may represent an advantage for early clinical decision-making. In addition, the inclusion of an initial heterogeneous state (A0) captures patients with less distinctive early trajectories and harder-to-predict outcomes, avoiding the need to force all patients into a fixed set of mutually exclusive phenotypes.

When comparing the identified sub-phenotypes across data sets, we observed a noteworthy level of alignment between cluster characteristics (Table 4). However, despite being generated from the same learned encoder, the clustering results still demonstrate the ability to reflect data set- or population-specific nuances. For example, Cluster A2 for AmsterdamUMCdb predominantly captures mechanically ventilated patients, while Cluster M2 for MIMIC-IV is more closely associated with patients experiencing kidney failure or requiring renal replacement therapy. Such differences highlight the adaptability of the clustering approach to the unique features of each data set. Importantly, while the overarching clinical patterns captured by the sub-phenotypes remain broadly comparable across the data sets, this approach preserves sufficient flexibility to capture specific clinical variations inherent to each population. This balance between generalisability and specificity underscores the potential utility of the clustering framework for heterogeneous cohorts, such as the population of sepsis patients.

Finally, our reinforcement learning-based evaluation provides a complementary perspective on how treatment patterns and associated outcomes vary across the identified sub-phenotypes. Rather than proposing prescriptive treatment recommendations, these results illustrate systematic differences in observed decision-making and outcome associations between patient subgroups. This echoes previous findings which demonstrated that data-driven sepsis sub-phenotypes could correlate with clinical outcomes [9], and reinforces the idea that more granular patient stratification could enhance precision in critical care.

### Limitations

This study has several limitations. First, the absence of a ground-truth classification for sepsis sub-phenotypes is, inherently, a complicating factor for validation. While external validation on the external MIMIC-IV database supports the robustness of our findings, further clinical validation would be necessary to assess the quality of the sub-phenotypes. Second, while we only considered an LSTM-based encoder for our model architecture, there are alternative architectures that may be superior for the encoding objective. For example, Rocheteau et al. found the temporal pointwise convolution (TPC) method to provide superior performance when compared to an LSTM model, for an encoding objective similar to our own [18]. In addition, incorporating the attention mechanism into our encoder model may help the encoder to capture long-term dependencies more effectively. However, the exploration of these ideas falls outside the scope of the current work. Third, while the present work demonstrates that the derived sub-phenotypes capture potentially clinically meaningful heterogeneity, translating these findings into actionable clinical insights would require prospective validation, potentially through decision support or simulation-based frameworks that build upon the reinforcement learning-based evaluation presented here. In addition, sub-phenotype structure may vary across patient populations and healthcare settings, which could influence treatment strategies and limit direct transferability of phenotype-specific insights between cohorts. Fourth, the temporal discretisation into fixed 4-h intervals represents a pragmatic design choice. While small shifts in window alignment may lead to minor differences in sub-phenotype assignment for individual patients, particularly near cluster-transition boundaries, we expect the dominant sub-phenotype structures to remain stable for the majority of patients, as clinically meaningful physiological changes typically evolve over longer timescales.

## Conclusions

Our findings demonstrate that data-driven sub-phenotyping can uncover meaningful and clinically interpretable patterns in sepsis, with potential implications for personalised treatment strategies. The identified sub-phenotypes generalise well across different data sets while accommodating population-specific variations. Furthermore, our reinforcement learning-based evaluation underscores the potential of sub-phenotypes to guide clinical decision-making, suggesting that tailored treatment strategies could improve patient outcomes. Future research should focus on prospective validation and clinical implementation to further assess the real-world impact of this phenotyping approach.

## Supplementary Information

**Additional file 1.**

## Data Availability

The data from AmsterdamUMCdb and MIMIC-IV are publicly available upon completion of the CITI Data or Specimens Only Research training. The source code used to produce the results and analyses presented in this manuscript is publicly available at https://github.com/paulhilders/SepsisPhenotyping.

## Abbreviations

AmsterdamUMCdb: Amsterdam University Medical Centres database
MIMIC: Medical Information Mart for Intensive Care
ICU: Intensive care unit
AI: Artificial intelligence
ML: Machine learning
LSTM: Long short-term memory
LOS: Length of stay
MV: Mechanical ventilation
RRT: Renal replacement therapy
SARSA: State–Action–Reward–State–Action
AST: Aspartate transaminase
ALT: Alanine transaminase
BUN: Blood urea nitrogen
CRP: C-reactive protein
INR: International normalised ratio
PaO_2_: Partial pressure of oxygen
PaCO_2_: Partial pressure of carbon dioxide
WBC: White blood cell count
SpO_2_: Oxygen saturation
CVP: Central venous pressure
GCS: Glasgow coma scale
PEEP: Positive end-expiratory pressure
IV: Intravenous
AUROC: Area under the receiver operating characteristic
MSE: Mean squared error
AKI: Acute kidney injury
LPA: Latent profile analysis
LCA: Latent class analysis
TPC: Temporal pointwise convolution
