# Supplementary material for: Prediction-guided clustering for sepsis phenotyping: a retrospective cohort analysis
Source: Intensive Care Med Exp. 2026 Mar 18;14:35. doi: 10.1186/s40635-026-00882-9 (PMC13000074; doi:10.1186/s40635-026-00882-9)
Supplement: Supplementary file 1 — Additional file 1. [file 40635_2026_882_MOESM1_ESM.docx]

## Appendix A Supplementary Material

### A.1 Sankey diagrams

Figure A1a and Figure A1b shows the cluster progression over time, for the first seven days in the AmsterdamUMCdb and MIMIC-IV cohorts. Two additional clusters were added to represent the mortality (purple) and discharged (pink) status.


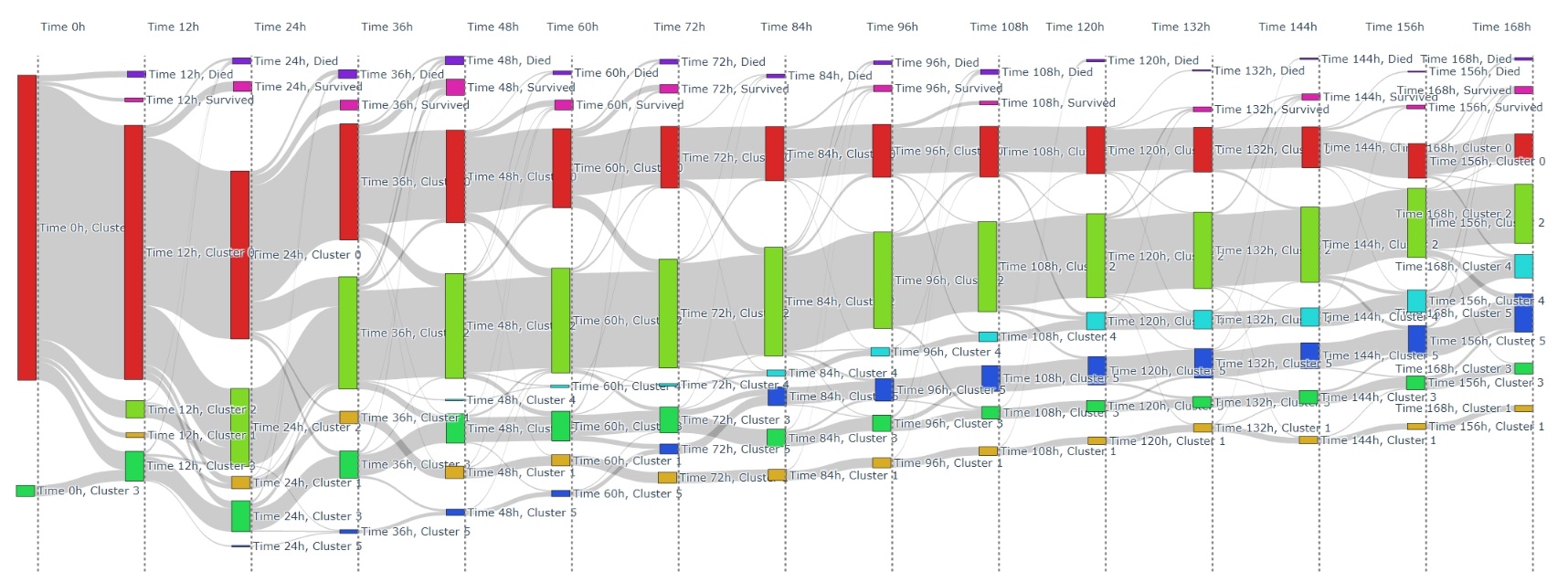


Figure A1a: Sankey diagram illustrating sub-phenotype progression during the first 7 ICU days in the AmsterdamUMCdb cohort. Each horizontal slice represents a consecutive 12-hour time window, with node width proportional to the number of patients assigned to each sub-phenotype. Flows indicate transitions between sub-phenotypes across adjacent windows, with thickness reflecting transition frequency. The figure is intended to convey dominant progression patterns rather than exact transition probabilities.


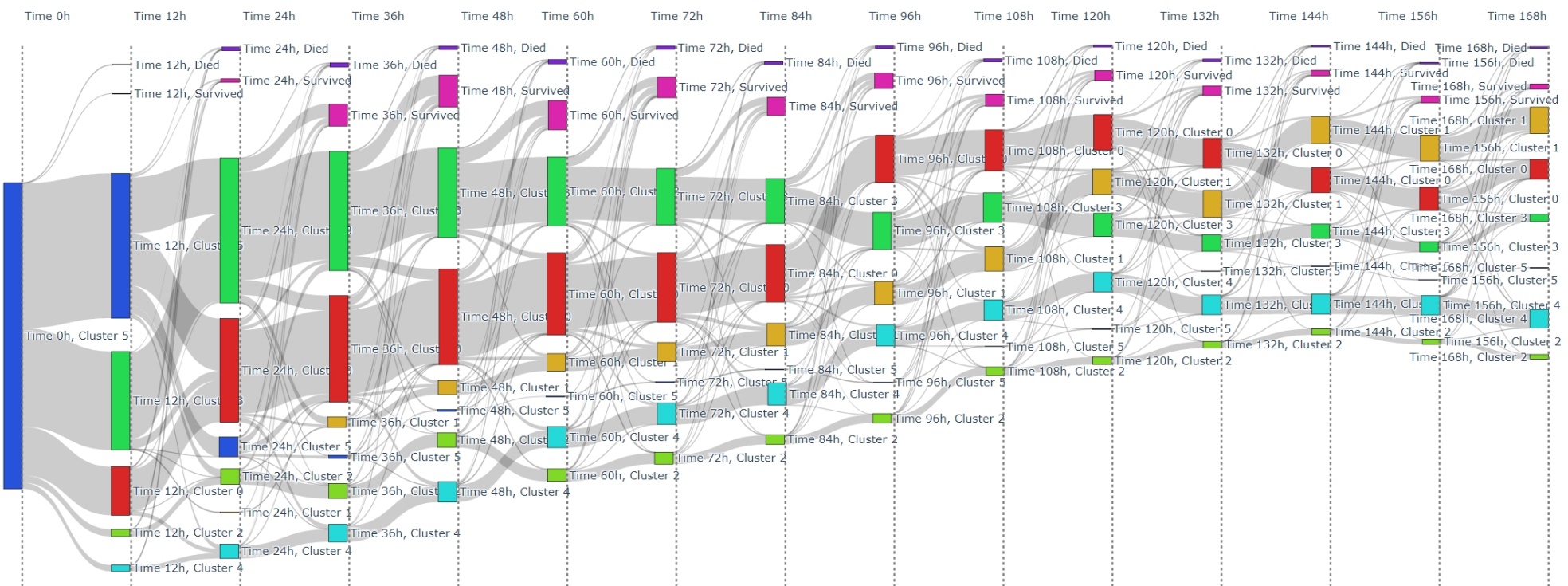


Figure A1b: Sankey diagram illustrating sub-phenotype progression during the first 7 ICU days in the MIMIC-IV cohort. Each horizontal slice represents a consecutive 12-hour time window, with node width proportional to the number of patients assigned to each sub-phenotype. Flows indicate transitions between sub-phenotypes across adjacent windows, with thickness reflecting transition frequency. The figure is intended to convey dominant progression patterns rather than exact transition probabilities.

### A.2 Sample statistics for MIMIC-IV

Table A2: Sample statistics across the six clusters from the MIMIC-IV validation set.

| **Features** | **Cohort** | **M0** | **M1** | **M2** | **M3** | **M4** | **M5** |
| --- | --- | --- | --- | --- | --- | --- | --- |
| **Number of time steps, N (%)** | 146026 | 34868 (23.9%) | 30242 (20.7%) | 7417 (5.1%) | 30953 (21.2%) | 26189 (17.9%) | 16357 (11.2%) |
| **AST, U/L** | 137.9 (±71.3) | 145.1 (±573.2) | 51.4 (±23.5) | 237.7 (±898.4) | 61.5 (±148.3) | 149.3 (±582.7) | 182.5 (±756.9) |
| **ALT, U/L** | 92.3 (±39.6) | 99.4 (±323.1) | 41.5 (±35.6) | 150.7 (±422.9) | 57.1 (±179.4) | 89.7 (±299.0) | 115.6 (±421.4) |
| **Bicarbonate, mmol/L** | 24.6 (±1.8) | 24.2 (±5.2) | 27.7 (±4.9) | 22.7 (±4.5) | 25.2 (±4.4) | 24.8 (±6.1) | 22.8 (±4.9) |
| **Total bilirubin, µmol/L** | 35.4 (±20.5) | 31.1 (±48.6) | 15.5 (±5.5) | 66.4 (±106.9) | 16.3 (±13.0) | 53.3 (±85.8) | 30.0 (±60.5) |
| **BUN, mmol/L** | 9.6 [7.3,14.4] | 10.4 [6.8,16.4] | 6.1 [3.9,9.3] | 16.4 [10.4,23.9] | 6.8 [4.3,10.0] | 15.7 [9.6,24.6] | 8.9 [6.4,15.4] |
| **Calcium, mmol/L** | 2.10 (±0.04) | 2.08 (±0.19) | 2.10 (±0.16) | 2.16 (±0.22) | 2.07 (±0.16) | 2.14 (±0.23) | 2.06 (±0.20) |
| **Chloride, mmol/L** | 103.0 (±1.4) | 104.1 (±6.7) | 103.2 (±6.0) | 100.8 (±6.7) | 104.4 (±6.0) | 102.2 (±6.7) | 103.6 (±6.6) |
| **Potassium, mmol/l** | 4.1 (±0.1) | 4.1 (±0.6) | 4.0 (±0.5) | 4.2 (±0.6) | 4.0 (±0.5) | 4.1 (±0.6) | 4.2 (±0.7) |
| **Serum creatinine, µmol/L** | 106.1 [84.0, 141.5] | 114.9 [79.6, 185.7] | 53.1 [44.2, 70.7] | 300.6 [159.2, 477.5] | 79.6 [53.1, 106.1] | 150.3 [97.3, 247.6] | 97.3 [79.6, 159.2] |
| **Blood glucose, mmol/L** | 7.1 [7.0,7.2] | 7.2 [6.0,9.1] | 6.9 [5.9,8.2] | 7.1 [5.8,9.3] | 6.8 [5.7,8.2] | 7.3 [6.1,9.2] | 7.0 [6.1,8.8] |
| **Heart rate, bpm** | 87.4 (±2.4) | 86.8 (±16.5) | 87.5 (±16.3) | 85.4 (±16.8) | 84.9 (±16.3) | 91.6 (±17.4) | 88.3 (±17.9) |
| **Hematocrit, fraction** | 0.291 (±0.014) | 0.294 (±0.051) | 0.290 (±0.048) | 0.275 (±0.045) | 0.305 (±0.052) | 0.275 (±0.044) | 0.306 (±0.060) |
| **Hemoglobin, g/L** | 5.9 (±0.3) | 6.0 (±1.1) | 5.8 (±1.0) | 5.6 (±0.9) | 6.2 (±1.1) | 5.5 (±0.9) | 6.2 (±1.2) |
| **INR** | 1.3 [1.3,1.3] | 1.3 [1.2,1.5] | 1.3 [1.2,1.3] | 1.3 [1.2,1.6] | 1.3 [1.2,1.3] | 1.3 [1.2,1.8] | 1.3 [1.2,1.5] |
| **Lactate, mmol/L** | 1.7 (±0.3) | 1.7 (±1.1) | 1.4 (±0.3) | 1.7 (±1.2) | 1.5 (±0.5) | 1.9 (±1.6) | 2.1 (±1.7) |
| **SpO_2_, %** | 97.2 [97.1,97.3] | 97.0 [95.2,98.8] | 97.5 [95.8,99.0] | 97.2 [95.7,99.0] | 96.8 [95.0,98.2] | 97.3 [95.5,99.0] | 97.2 [95.5,99.0] |
| **PaO_2_, mmHg** | 95.0 [95.0, 95.0] | 95.0 [82.0, 106.0] | 95.0 [95.0, 96.0] | 95.0 [83.0, 102.7] | 95.0 [89.0, 104.0] | 95.0 [79.0, 106.0] | 95.0 [75.0, 125.0] |
| **PaCO_2_, mmHg** | 41.0 [41.0, 41.0] | 41.0 [38.0, 43.0] | 41.0 [41.0, 41.0] | 41.0 [37.0, 41.0] | 41.0 [38.0, 41.0] | 41.0 [39.0, 45.5] | 41.0 [37.5, 43.2] |
| **Arterial pH** | 7.40 [7.40,7.40] | 7.40 [7.37,7.42] | 7.40 [7.40,7.44] | 7.40 [7.37,7.42] | 7.40 [7.40,7.43] | 7.40 [7.36,7.42] | 7.40 [7.34,7.40] |
| **Platelets** | 173.0 [146.0, 196.2] | 158.0 [101.0, 227.0] | 267.0 [188.0, 384.0] | 142.0 [75.0, 222.0] | 199.0 [142.0, 273.0] | 141.0 [70.0, 229.0] | 188.0 [124.0, 239.0] |
| **Respiratory rate, per minute** | 20.5 (±0.7) | 20.4 (±5.0) | 20.9 (±5.0) | 20.3 (±5.2) | 19.7 (±4.7) | 21.6 (±5.5) | 20.1 (±4.8) |
| **Sodium, mmol/L** | 139.2 (±0.7) | 139.5 (±5.7) | 140.3 (±5.0) | 138.2 (±5.1) | 139.2 (±4.9) | 139.5 (±5.7) | 138.6 (±5.3) |
| **Systolic BP, mmHg** | 119.1 (±4.7) | 119.5 (±19.6) | 124.8 (±19.4) | 120.8 (±20.2) | 122.3 (±18.9) | 112.5 (±17.7) | 114.7 (±18.4) |
| **Mean BP, mmHg** | 78.1 (±3.8) | 77.0 (±12.6) | 83.9 (±13.4) | 78.0 (±14.2) | 80.6 (±12.8) | 72.9 (±12.4) | 76.3 (±12.5) |
| **Diastolic BP, mmHg** | 61.9 (±3.7) | 60.2 (±11.9) | 67.7 (±12.4) | 61.1 (±13.7) | 64.3 (±12.2) | 56.7 (±11.5) | 61.6 (±12.1) |
| **Temperature (ºC)** | 36.9 (±0.1) | 37.0 (±0.6) | 37.1 (±0.6) | 36.9 (±0.6) | 37.0 (±0.6) | 36.8 (±0.6) | 36.8 (±0.7) |
| **WBC. x 10^9^/L** | 11.1 [10.7, 11.3] | 11.4 [8.2, 15.7] | 10.5 [8.0, 13.7] | 11.0 [7.7, 15.5] | 10.6 [7.9, 13.9] | 12.3 [8.7, 17.7] | 11.2 [8.4, 15.7] |
| **GCS eye** | 3.3 (±0.2) | 3.0 (±1.2) | 3.4 (±1.0) | 3.3 (±1.0) | 3.5 (±0.9) | 3.1 (±1.1) | 3.3 (±1.1) |
| **GCS motor** | 5.2 (±0.3) | 4.9 (±1.7) | 5.4 (±1.2) | 5.2 (±1.5) | 5.7 (±0.9) | 4.7 (±1.8) | 5.2 (±1.6) |
| **GCS verbal** | 4.5 (±0.2) | 4.3 (±1.3) | 4.5 (±1.1) | 4.5 (±1.0) | 4.8 (±0.6) | 4.3 (±1.4) | 4.7 (±0.8) |
| **Age (norm)** | 0.27 (±0.13) | 0.38 (±0.50) | 0.08 (±0.54) | 0.13 (±0.50) | 0.28 (±0.51) | 0.40 (±0.49) | 0.34 (±0.52) |
| **Weight (norm)** | 0.03 (±0.17) | -0.03 (±0.77) | 0.13 (±0.93) | 0.34 (±0.98) | -0.01 (±0.84) | -0.13 (±0.71) | -0.10 (±0.78) |
| **Height (norm)** | -0.26 (±0.06) | -0.28 (±0.44) | -0.24 (±0.50) | -0.14 (±0.45) | -0.29 (±0.46) | -0.31 (±0.44) | -0.28 (±0.42) |
| **Weight missing, N (%)** | 1129 (0.8%) | 264 (0.8%) | 54 (0.2%) | 31 (0.4%) | 199 (0.6%) | 318 (1.2%) | 234 (1.4%) |
| **Height missing, N (%)** | 42386 (29.0%) | 11658 (33.4%) | 5514 (18.2%) | 1645 (22.2%) | 8705 (28.1%) | 8003 (30.6%) | 6810 (41.6%) |
| **Gender (male), N (%)** | 85789 (58.7%) | 20514 (58.8%) | 17268 (57.1%) | 5072 (68.4%) | 17322 (56.0%) | 14388 (54.9%) | 9369 (57.3%) |
| **90-day mortality, N (%)** | 32147 (22.0%) | 8286 (23.8%) | 3647 (12.1%) | 1692 (22.8%) | 3261 (10.5%) | 11194 (42.7%) | 3300 (20.2%) |
| **LOS outcome, days** | 3.3 [3.0, 3.8] | 3.3 [1.1, 7.7] | 4.0 [1.3, 9.0] | 3.3 [1.0, 8.1] | 2.2 [0.6, 6.0] | 5.3 [1.8, 11.8] | 2.9 [1.3, 6.4] |
| **RRT outcome, N (%)** | 13639 (9.3%) | 1721 (4.9%) | 192 (0.6%) | 1991 (26.8%) | 114 (0.4%) | 5384 (20.6%) | 442 (2.7%) |
| **MV outcome, N (%)** | 62502 (42.8%) | 15807 (45.3%) | 13808 (45.7%) | 2912 (39.3%) | 9130 (29.5%) | 14686 (56.1%) | 6704 (41.0%) |
| *Values are mean(±SD), median[Q1,Q3] or N (%).*  *AST – aspartate aminotransferase, ALT – alanine aminotransferase, BUN – blood urea nitrogen, INR – international normalized ratio, BP – blood pressure, WBC – white blood cell count, GCS – Glasgow coma score, LOS – length of stay, RRT – renal replacement therapy, MV – mechanical ventilation* | | | | | | | |

### A.3 Attribution maps for MIMIC-IV


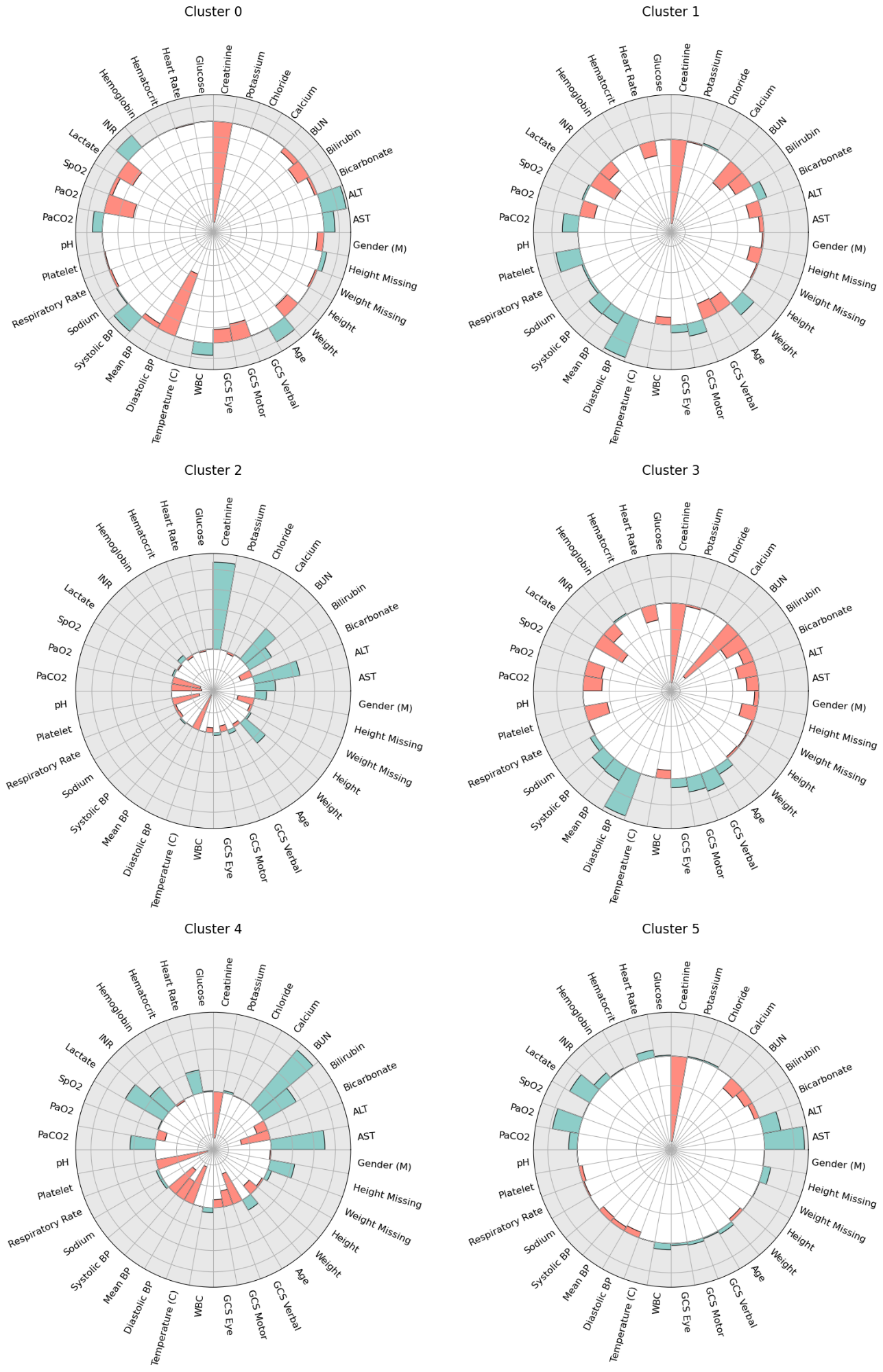


Figure A3: A visualisation of Integrated Gradients-based attribution values across the various features and clusters in the MIMIC-IV dataset. A larger value or bar size represents a relatively large contribution of that feature to the average encoding that was assigned to the corresponding cluster. The colour and directionality of the bar denote a high (green/outside) or low (red/inside) average feature value, relative to the other clusters.

### A.4 Hyperparameter configuration

In this section, we provide an overview of the hyperparameter configuration used in this study.

#### A.4.1 Network architecture

- **Model type:** Long Short-Term Memory (LSTM)
- **Encoder architecture:** 3 LSTM layers
- **Encoding size:** 32
- **Predictor head architecture:** MLP with one hidden layer, batch normalisation and a ReLU activation function. The hidden layer dimensionality was 16. For output activations, we used an exponential activation function for the Length-Of-Stay prediction, and a linear activation for the other heads.

#### A.4.2 Training Procedure

- **Loss functions:** We used the Binary Cross Entropy loss for the binary prediction heads and the Mean Squared Error loss for the Length-Of-Stay prediction.
- **Optimiser:** Adam
- **Learning rate:** 0.01
- **Validation set fraction:** 15%
- **Test set fraction:** 15%
- **Training epochs:** We trained for a maximum of 200 epochs, but performed early stopping after validation loss stopped improving over 5 epochs.
- **Batch size:** 256
- **Hardware used:** NVIDIA GeForce RTX 2060 SUPER GPU

#### A.4.3 Implementation-specific aspects

- **Framework:** PyTorch version 2.4.1

### A.5 Reinforcement learning evaluation – Action space design

In this section, we provide the action space definition used in our reinforcement learning evaluation (Table A5). To ensure comparability with related studies, we adopted the action space design from [14]. The discretised categories represent increasing levels of treatment intensity for both fluid resuscitation and vasopressor support, ranging from no intervention to progressively higher dosing levels. These bins are intended to reflect clinically plausible escalation steps commonly observed in ICU practice rather than optimised treatment strategies.

Table A5: Definition of the discretised action space used in the reinforcement learning analysis. Action categories are adopted from [14] and represent clinically plausible treatment options rather than optimised strategies.

|  | IV fluids (mL/4 hours) | Vasopressors (mcg/kg/min) |
| --- | --- | --- |
| Discretised Action | **Range** | **Range** |
| 1 | 0 | 0 |
| 2 | *]0-50]* | *]0-0.08]* |
| 3 | *]50-180]* | *]0.08-0.22]* |
| 4 | *]180-530]* | *]0.22-0.45]* |
| 5 | *>530* | *>0.45* |

### A.6 Determination of the optimal number of clusters

To determine the optimal number of clusters, we performed silhouette analysis on the validation dataset after encoding patient trajectories using the trained encoder. The silhouette score quantifies how well each sample fits within its assigned cluster relative to neighbouring clusters, with higher values indicating better separation.

Candidate cluster numbers were evaluated across a predefined range from k = 2 to k = 10 , using identical encoded representations and clustering settings. Based on this analysis, k = 6 was selected as providing a favourable balance between cluster separation and interpretability.
